# Development and Validation of Automated Magnetic Resonance Parkinsonism Index 2.0 to Distinguish Progressive Supranuclear Palsy‐Parkinsonism From Parkinson's Disease

**DOI:** 10.1002/mds.28992

**Published:** 2022-04-11

**Authors:** Andrea Quattrone, Maria G. Bianco, Angelo Antonini, David E. Vaillancourt, Klaus Seppi, Roberto Ceravolo, Antonio P. Strafella, Gioacchino Tedeschi, Alessandro Tessitore, Roberto Cilia, Maurizio Morelli, Salvatore Nigro, Basilio Vescio, Pier Paolo Arcuri, Rosa De Micco, Mario Cirillo, Luca Weis, Eleonora Fiorenzato, Roberta Biundo, Roxana G. Burciu, Florian Krismer, Nikolaus R. McFarland, Christoph Mueller, Elke R. Gizewski, Mirco Cosottini, Eleonora Del Prete, Sonia Mazzucchi, Aldo Quattrone

**Affiliations:** ^1^ Institute of Neurology, University “Magna Graecia” Catanzaro Italy; ^2^ Department of Clinical and Movement Neurosciences, UCL Queen Square Institute of Neurology University College London London United Kingdom; ^3^ Department of Medical and Surgical Sciences University “Magna Graecia” Catanzaro Italy; ^4^ Neuroscience Research Center University “Magna Graecia” Catanzaro Italy; ^5^ Parkinson and Movement Disorders Unit, Study Center for Neurodegeneration CESNE, Department of Neuroscience University of Padua Padua Italy; ^6^ Department of Applied Physiology and Kinesiology University of Florida Gainesville Florida USA; ^7^ Department of Neurology and Biomedical Engineering University of Florida Gainesville Florida USA; ^8^ Department of Neurology Medical University Innsbruck Innsbruck Austria; ^9^ Neuroimaging Core Facility Medical University Innsbruck Innsbruck Austria; ^10^ Department of Clinical and Experimental Medicine, Center for NeuroDegenerative Diseases University of Pisa Pisa Italy; ^11^ Krembil Brain Institute, UHN & Research Imaging Center, Campbell Family Mental Health Research Institute, CAMH University of Toronto Toronto Ontario Canada; ^12^ Department of Advanced Medical and Surgical Sciences University of Campania “Luigi Vanvitelli” Naples Italy; ^13^ MRI Research Center SUN‐FISM University of Campania “Luigi Vanvitelli” Naples Italy; ^14^ Department of Clinical Neurosciences, Fondazione IRCCS Istituto Neurologico Carlo Besta Parkinson and Movement Disorders Unit Milan Italy; ^15^ Institute of Nanotechnology (NANOTEC) National Research Council Lecce Italy; ^16^ Center for Neurodegenerative Diseases and the Aging Brain, Department of Clinical Research in Neurology University of Bari Aldo Moro, "Pia Fondazione Cardinale G. Panico" Tricase Italy; ^17^ Institute of Molecular Bioimaging and Physiology National Research Council (IBFM‐CNR) Catanzaro Italy; ^18^ Department of Radiology Pugliese‐Ciaccio Hospital Catanzaro Italy; ^19^ Department of General Psychology University of Padua Padua Italy; ^20^ Department of Kinesiology and Applied Physiology University of Delaware Newark Delaware USA; ^21^ Department of Neuroradiology Medical University Innsbruck Innsbruck Austria; ^22^ Department of Translational Research and New Technologies University of Pisa Pisa Italy

**Keywords:** Magnetic Resonance Parkinsonism Index 2.0, progressive supranuclear palsy‐parkinsonism, Parkinson's disease, automated MRI biomarker

## Abstract

**Background:**

Differentiating progressive supranuclear palsy‐parkinsonism (PSP‐P) from Parkinson's disease (PD) is clinically challenging.

**Objective:**

This study aimed to develop an automated Magnetic Resonance Parkinsonism Index 2.0 (MRPI 2.0) algorithm to distinguish PSP‐P from PD and to validate its diagnostic performance in two large independent cohorts.

**Methods:**

We enrolled 676 participants: a training cohort (n = 346; 43 PSP‐P, 194 PD, and 109 control subjects) from our center and an independent testing cohort (n = 330; 62 PSP‐P, 171 PD, and 97 control subjects) from an international research group. We developed a new in‐house algorithm for MRPI 2.0 calculation and assessed its performance in distinguishing PSP‐P from PD and control subjects in both cohorts using receiver operating characteristic curves.

**Results:**

The automated MRPI 2.0 showed excellent performance in differentiating patients with PSP‐P from patients with PD and control subjects both in the training cohort (area under the receiver operating characteristic curve [AUC] = 0.93 [95% confidence interval, 0.89–0.98] and AUC = 0.97 [0.93–1.00], respectively) and in the international testing cohort (PSP‐P versus PD, AUC = 0.92 [0.87–0.97]; PSP‐P versus controls, AUC = 0.94 [0.90–0.98]), suggesting the generalizability of the results. The automated MRPI 2.0 also accurately distinguished between PSP‐P and PD in the early stage of the diseases (AUC = 0.91 [0.84–0.97]). A strong correlation (*r* = 0.91, *P* < 0.001) was found between automated and manual MRPI 2.0 values.

**Conclusions:**

Our study provides an automated, validated, and generalizable magnetic resonance biomarker to distinguish PSP‐P from PD. The use of the automated MRPI 2.0 algorithm rather than manual measurements could be important to standardize measures in patients with PSP‐P across centers, with a positive impact on multicenter studies and clinical trials involving patients from different geographic regions. © 2022 The Authors. *Movement Disorders* published by Wiley Periodicals LLC on behalf of International Parkinson and Movement Disorder Society

The clinical differential diagnosis between progressive supranuclear palsy‐parkinsonism (PSP‐P) and Parkinson's disease (PD) may be challenging, especially in the first years after the disease onset.^1^, ^2^, ^3^, ^4^ Patients with PSP‐P have a clinical phenotype mainly characterized by parkinsonism, which can be asymmetric and levodopa‐responsive, strongly resembling PD.^2^, ^3^, ^4^, ^5^, ^6^, ^7^, ^8^, ^9^ The only clinical sign specific for PSP is the ocular motor dysfunction, but the vertical supranuclear gaze palsy may appear up to 19 years after disease onset, making diagnosis at times difficult.^6^, ^7^, ^8^, ^9^

To date, several imaging biomarkers have proved to be useful in distinguishing PSP from PD, and the Magnetic Resonance Parkinsonism Index (MRPI) is one of the most powerful and robust ones.^10^, ^11^, ^12^, ^13^, ^14^, ^15^, ^16^, ^17^, ^18^, ^19^, ^20^, ^21^ The large majority of these imaging biomarkers, however, showed high diagnostic accuracy for PSP‐Richardson's syndrome (PSP‐RS) but failed to accurately distinguish patients with PSP‐P from patients with PD, probably because of the lower degree of brain atrophy in this milder PSP subtype.^5^, ^10^, ^13^ A new version of the MRPI (termed MRPI 2.0) has been recently developed to overcome this limitation.^22^ In addition to the brainstem structures measured by MRPI (midbrain, pons, middle and superior cerebellar peduncles), MRPI 2.0 also includes the measurement of the third ventricle (3 V) width, a brain structure that is commonly enlarged in patients with PSP but spared in patients with PD.^22^, ^23^, ^24^

Preliminary results have shown that MRPI 2.0 accurately differentiated patients with PSP‐P from patients with PD,^22^ but this finding was not confirmed in a more recent single‐center study,^18^ where this biomarker was less accurate in distinguishing PSP‐P from PD probably because of the variability of manual measurements and small sample size, thus suggesting the need for automated MRPI 2.0 calculation and for validation studies in larger international cohorts.

The aim of this study was the development of a fully automated algorithm to calculate MRPI 2.0 and the validation of the automated MRPI 2.0 performance in differentiating patients with PSP‐P from patients with PD and control subjects in two large independent cohorts from different geographic regions.

## Subjects and Methods

### Patients

A total of 676 participants were enrolled in this study, divided into a training and a testing cohort. The training cohort included 346 participants (43 PSP‐P, 194 PD, and 109 control subjects) from our center, while the independent testing cohort included 330 participants (62 PSP‐P, 171 PD, and 97 control subjects) from an international research group.

Participants in the training cohort were consecutively recruited between March 2012 and January 2020 at the Movement Disorder Center of Magna Graecia University, Catanzaro, Italy. The PSP‐P, PD, and control subjects included in the testing cohort were enrolled from seven different centers (Supporting Information Table S1). The diagnoses of PD and PSP‐P were performed by movement disorder specialists using international clinical diagnostic criteria.^6^, ^25^ Patients with PSP‐P enrolled before 2017 were diagnosed according to expert guidelines^7^ and were retrospectively reclassified according to recent Movement Disorder Society (MDS) diagnostic criteria for probable PSP‐P (vertical ocular dysfunction associated with parkinsonism as predominant clinical features in the absence of early falls).^6^, ^26^ All patients underwent a neurological examination, including the MDS‐sponsored revision of the Unified Parkinson's Disease Rating Scale part III (MDS‐UPDRS‐III),^27^ in *off* state and the Hoehn and Yahr (H‐Y) rating scale.^28^

Exclusion criteria for patients with PD and PSP consisted of age <40 years, clinical features suggestive of other diseases, normal striatal uptake on ^123^I‐ioflupane Single Photon Emission Computed Tomography (DaTscan), and magnetic resonance imaging (MRI) abnormalities such as lacunar infarctions in the basal ganglia and/or subcortical vascular lesions with diffuse periventricular signal alterations. None of the control participants were younger than 40 years or had a history of neurological, psychiatric, or other major medical illnesses. We also excluded subjects from the study who showed Evans Index >0.32 associated with callosal angle <100 degrees, which is a combination of MRI biomarkers strongly suggestive of normal pressure hydrocephalus.^29^ A percentage of patients included in the current cohorts (56 patients with PSP‐P, 270 patients with PD, and 139 control subjects) have been reported in a recent study to validate the automated MRPI,^14^ but the MRPI 2.0 was not tested in this previous study.

All study procedures and ethical aspects were approved by an institutional review board (Magna Graecia University review board, Catanzaro, Italy). Each recruitment site received approval from an institutional review board or ethics committee. Written informed consent according to the Declaration of Helsinki for the use of their medical records for research purposes was obtained from all individuals participating in the study.

### MRI Protocol

All patients and controls in the training cohort underwent a brain MRI with a 3‐T MR750 General Electric scanner and an eight‐channel head coil, with a recently described MRI protocol.^22^

Patients and control subjects from the international cohort underwent a brain MRI with 3‐T (62 PSP‐P, 145 PD, and 80 control subjects) or 1.5‐T scanners (26 PD and 17 control subjects), with a protocol including a T1‐weighted volumetric image. The 3D T1‐weighted MRIs were uploaded on the web‐based platform for the automated MRPI calculation (https://mrpi.unicz.it)^14^ by all the international research centers.

### MRPI 2.0 Calculation

**FIG 1:**
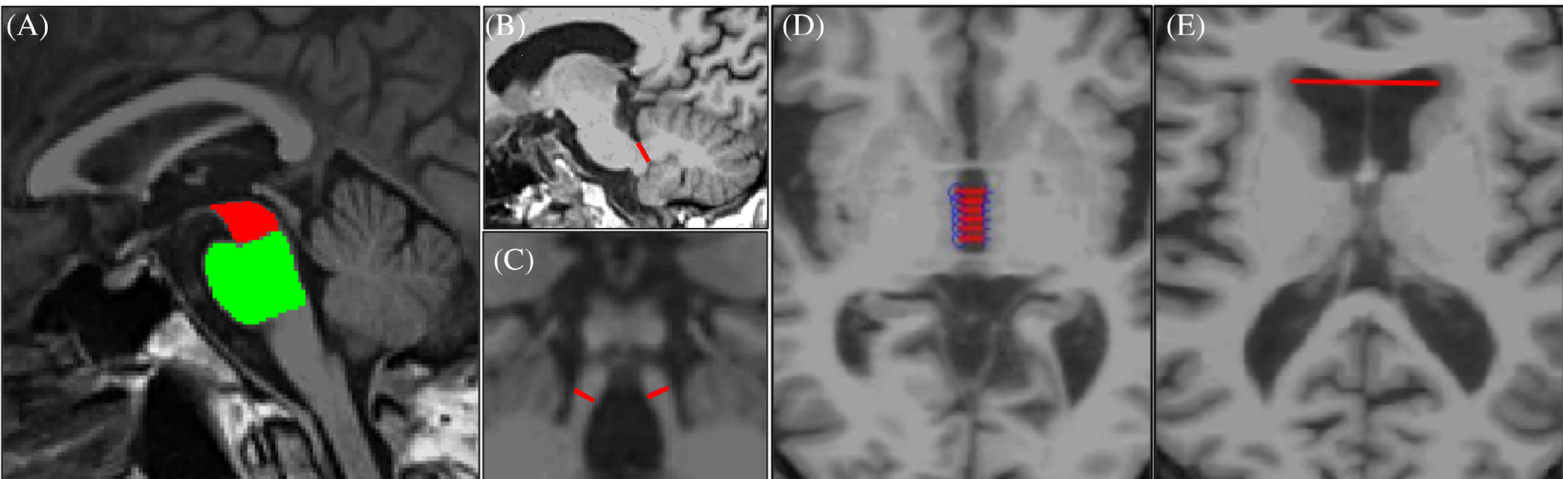
Automated measurement of the midbrain and pons area (A), the middle cerebellar peduncle width (B), the superior cerebellar peduncles width (C), the third ventricle width (D), and the maximum frontal horns width (E) on T1‐weighted magnetic resonance images. [Color figure can be viewed at wileyonlinelibrary.com]

The automated MRPI 2.0 was obtained by multiplying the automated MRPI value by the automated 3 V width/frontal horns (FHs) width ratio (Fig. 1). The automated MRPI value was calculated using the previously described toolbox.^11^, ^30^ The pipeline for the automated measurement of the 3 V width and FHs width is shown in Supporting Information Figure S1. The fully automated toolbox for MRPI 2.0 calculation was in‐house developed using R2017a MATLAB software. The proposed segmentation framework is based on the combined use of an anatomical landmark‐based approach and a thresholding‐based method.^31^ Specifically, T1‐weighted structural MRIs were normalized into a Montreal Neurological Institute template (six‐parameter affine registration) using FSL software (FMRIB Software Library). Intensity normalization of the T1‐weighted images was performed as previously described.^30^ Subsequently, the midsagittal plane was automatically defined using as anatomical landmarks the corpus callosum, the upper part of the brainstem, and the maximal expansion of Sylvius aqueduct.^30^ The deterministic algorithm based on a threshold approach for identifying midsagittal slice was that described by Nigro et al^30^ with some modifications (Supporting Information). Subsequently, a reformatted volumetric slab (including 35 slices each with 1 mm thickness) parallel to subcallosal line was generated to expose several axial views of the 3 V and the FHs of the lateral ventricles. In each axial slice showing the 3 V, the algorithm performed two automated linear measurements between its lateral borders and identified the slice with the largest 3 V width. Subsequently, in this selected slice, the 3 V width was calculated as the mean of six automated linear measures of the distance between its lateral borders, and this value was used for MRPI 2.0 calculation. Finally, the FHs of lateral ventricles were automatically segmented using anatomical and threshold approaches (Supporting Information). For each axial slice, the largest left‐to‐right width of FHs was measured, and the maximum value was used for MRPI 2.0 calculation. All automated segmentations were visually inspected to be sure that the brain structures identified by the automated procedure were correct. Automated MRPI 2.0 calculation was performed twice on the same MRIs in a subgroup of 30 participants (10 PSP‐P, 10 PD, and 10 control subjects) to assess the reproducibility of the automated method. Manual measurements of MRPI 2.0 were performed according to previously described procedures^22^ in a subgroup of 250 participants (50 PSP‐P, 100 PD, and 100 control subjects) by one expert rater who was blinded to clinical diagnosis and automated MR measures, and the correlation between automated and manual MRPI 2.0 values was investigated.

### Statistical Analysis

Difference in sex distribution was assessed by Fisher's exact test. The Shapiro–Wilk test was used to check for normality to decide whether parametric or nonparametric tests were appropriate for comparisons. Age at examination was compared using Kruskal–Wallis test followed by pairwise Wilcoxon rank sum test; age at disease onset and disease duration were compared using Wilcoxon rank sum test. Differences in MDS‐UPDRS‐III score, H‐Y score, MRPI, 3 V, FHs, 3 V/FHs ratio, and MRPI 2.0 values were investigated using analysis of covariance on generalized linear models with age and sex as covariates, followed by the Tukey test. The scanner field strength (1.5 and 3.0 T) was also included as covariate in the testing cohort; the MDS‐UPDRS‐III score was also included as covariate in the PSP‐P versus PD comparison of imaging data in both cohorts. All tests were 2‐tailed, and the α level was set at *P* < 0.05. All *P* values were corrected according to Bonferroni. We assessed the diagnostic performance of the automated MRPI and MRPI 2.0 in differentiating patients with PSP‐P from patients with PD and control subjects, in both the training and testing cohorts. Optimal cutoffs, defined as the values with the highest sum of sensitivity and specificity on the receiver operating characteristic (ROC) curves, and 95% confidence intervals (CIs) were calculated using pROC software package with bootstrapping (n = 2000 iterations).^32^ The diagnostic performances of MRPI 2.0 in the two cohorts were compared using the De Long test, to assess the generalizability of the findings.

We also investigated the MRPI 2.0 diagnostic performances after excluding the possible age effect. To correct for age effect, we fitted a linear regression model on MRPI 2.0 values in control subjects, both in the in‐house and in the external cohort, and computed residuals for PD, PSP‐P, and control subjects in both cohorts. Logistic regression models were evaluated for PSP‐P versus PD and PSP‐P versus controls classification, using both original MRPI 2.0 data and residuals. For each model, a ROC analysis was performed, and the De Long test was used to compare ROC curves evaluated on raw MRPI 2.0 values to ROC curves evaluated on residuals. The effect of sex on MRPI 2.0 classification of PSP‐P was evaluated in male and female subjects separately.

Logistic regression models were also used to evaluate the association between MRPI 2.0 values and the probability of having PSP‐P in both cohorts, taking into account the proportion between patients with PD and PSP‐P. The correlation between automated and manual MRPI 2.0 values was evaluated using Pearson's correlation test. The intraclass correlation coefficient was calculated to investigate the reproducibility of automated MRPI 2.0 measurements. Statistical analyses were performed using R statistical software (R for Unix/Linux, version 3.1.1; the R Foundation for Statistical Computing, 2014) and the ROCR package for R.

## Results

In this study, we developed a fully automated algorithm to calculate the MRPI 2.0. This software provided the calculation in 92.5% of cases (625/676 MRI DICOM images), and failure occurred in only 7 patients with PSP‐P, 21 patients with PD, and 23 control subjects, who were excluded from the subsequent analyses. Thirty of the failures were due to errors in the automated MRPI calculation, while the remaining 21 failures were due to errors in the automated measurement of the 3 V or FHs width, probably caused by motion artefacts that did not allow the automatic identification of anatomical landmarks used for the segmenting procedures.

The final training cohort included 312 patients from our center of Catanzaro, Italy (43 patients with PSP‐P, 177 patients with PD, and 92 control subjects), while the independent testing cohort included 313 patients from several international research groups (56 patients with PSP‐P, 166 patients with PD, and 91 control subjects). The demographic, clinical, and imaging data of patients and control subjects in the two cohorts are summarized in Table 1. In both cohorts, patients with PSP‐P were significantly older than patients with PD and control subjects; thus, all analyses were corrected for age at examination. Patients with PSP‐P had similar disease duration but higher disease severity in comparison with patients with PD in both the training and testing cohorts (Table 1).

**TABLE 1:** Demographic, clinical, and imaging data of patients with progressive supranuclear palsy‐parkinsonism, patients with Parkinson's disease, and control subjects in the training and testing cohorts

	Training Cohort				Testing Cohort			
Data	PSP‐P (n = 43)	PD (n = 177)	Control Subjects (n = 92)	*P* Value	PSP‐P (n = 56)	PD (n = 166)	Control Subjects (n = 91)	*P* Value
Sex (M/F)	29/14	104/73	45/47	0.113^a^	37/19	100/66	47/44	0.200^a^
Age at examination, y (mean ± SD)	71.7 ± 5.5^b^ ^,^ ^c^	65.8 ± 8.4^d^	63.2 ± 8.5	<0.001^e^	70.6 ± 6.1^b^ ^,^ ^c^	64.7 ± 9.5	63.7 ± 8.9	<0.001^e^
Age at disease onset, y (mean ± SD)	65.7 ± 6.2^b^	59.7 ± 8.6	/	<0.001^f^	66.8 ± 6.5^b^	59.4 ± 9.6	/	<0.001^f^
Disease duration, y (mean ± SD)	5.9 ± 3.5	6.1 ± 3.8	/	0.570^f^	4.1 ± 2.2	5.3 ± 4.1	/	0.240^f^
MDS‐UPDRS‐III score, median (range)	37 (16–55)^b^	27 (6–66)	/	<0.001^g^	40 (15–64)^b^	24 (4–68)	/	<0.001^g^
H‐Y score, median (range)	3 (2–5)^b^	2 (1–4)	/	<0.001^g^	3 (2–5)^b^	2 (1–4)	/	<0.001^g^
Brain MRI automated measurements								
MRPI (mean ± SD)	14.28 ± 3.98^b^ ^,^ ^c^	9.90 ± 3.35	9.48 ± 1.81	<0.001^g^	16.53 ± 5.98^b^ ^,^ ^c^	10.25 ± 2.26	9.80 ± 2.19	<0.001^g^
3 V width, mm (mean ± SD)	8.61 ± 2.44^b^ ^,^ ^c^	5.17 ± 2.10	4.29 ± 1.70	<0.001^g^	9.79 ± 2.62^b^ ^,^ ^c^	5.99 ± 2.23	5.32 ± 2.16	<0.001^g^
3 V/FHs ratio (mean ± SD)	0.22 ± 0.05^b^ ^,^ ^c^	0.15 ± 0.05^d^	0.12 ± 0.04	<0.001^g^	0.25 ± 0.05^b^ ^,^ ^c^	0.16 ± 0.05	0.14 ± 0.05	<0.001^g^
MRPI 2.0 (mean ± SD)	3.25 ± 1.32^b^ ^,^ ^c^	1.48 ± 0.83	1.20 ± 0.53	<0.001^g^	4.26 ± 2.16^b^ ^,^ ^c^	1.65 ± 0.76	1.47 ± 0.72	<0.001^g^

The disease onset was defined as the onset of the first PSP‐related symptom (motor or nonmotor), according to the Movement Disorder Society criteria for PSP clinical diagnosis.

PSP‐P, progressive supranuclear palsy‐parkinsonism; PD, Parkinson's disease; M, male; F, female; MDS‐UPDRS‐III, Movement Disorder Society–Unified Parkinson's Disease Rating Scale Part III (motor examination); H‐Y, Hoehn and Yahr rating scale; MRI, magnetic resonance imaging; MRPI, Magnetic Resonance Parkinsonism Index; 3 V, third ventricle; FH, frontal horn.

^a^ Fisher's exact test.

^b^ *P* < 0.001 (PSP‐P versus PD).

^c^ *P* < 0.001 (patients versus control subjects).

^d^ *P* < 0.05 (patients versus control subjects).

^e^ Kruskal–Wallis test followed by pairwise Wilcoxon rank sum test.

^f^ Wilcoxon rank sum test.

^g^ Analysis of covariance with age and sex as covariates, followed by Tukey test. The scanner field strength (1.5 and 3.0 T) was also included as covariate in the testing cohort; the MDS‐UPDRS‐III score was also included as covariate in the PSP‐P versus PD comparison in both cohorts. All *P* values were corrected according to Bonferroni.

**FIG 2:**
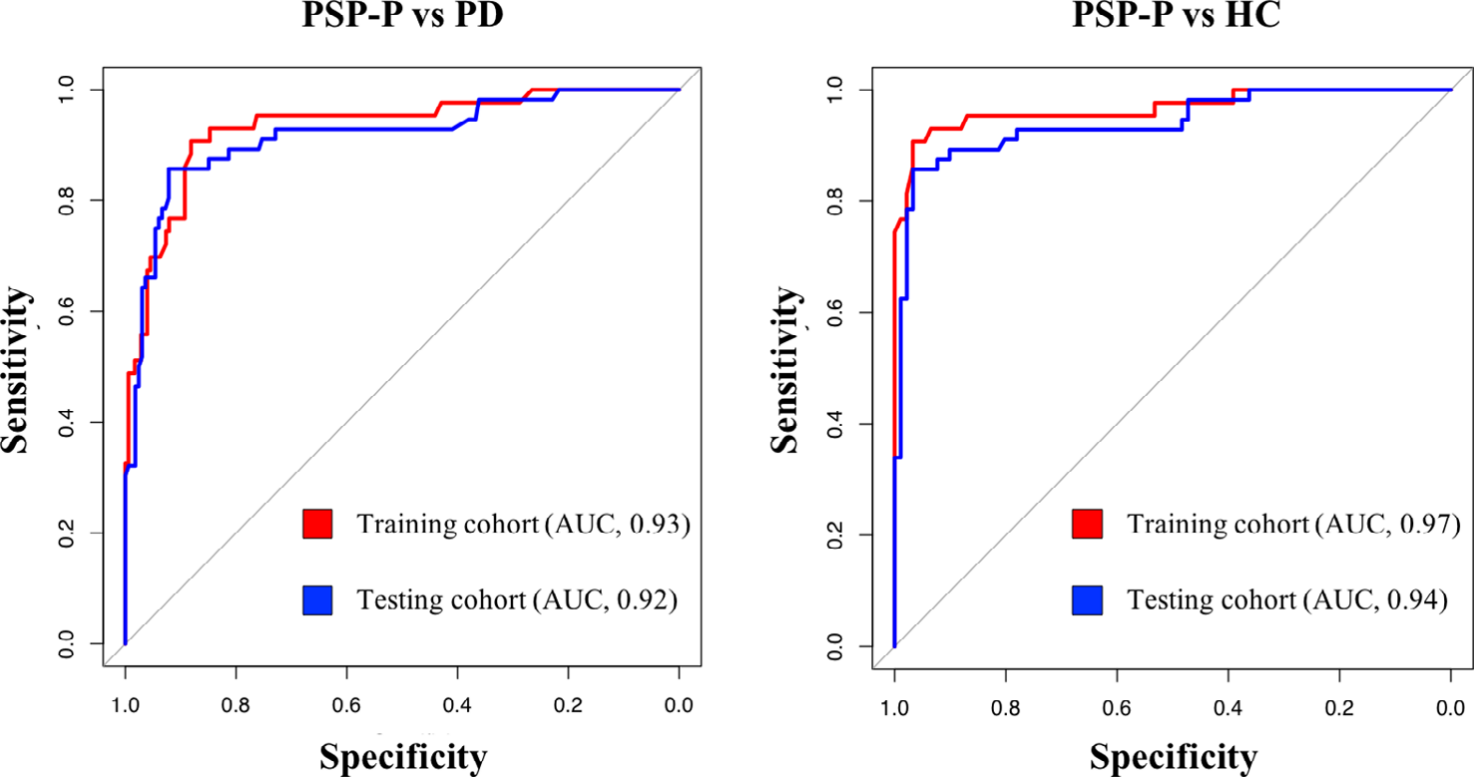
Receiver operating characteristic (ROC) curves for assessing the classification performance of automated Magnetic Resonance Parkinsonism Index 2.0 (MRPI 2.0) in differentiating (A) patients with progressive supranuclear palsy‐parkinsonism (PSP‐P) from patients with Parkinson's disease (PD), and (B) patients with PSP‐P from control subjects in the training (red) and testing (blue) cohorts. AUC, area under the ROC curve. [Color figure can be viewed at wileyonlinelibrary.com]

**FIG 3:**
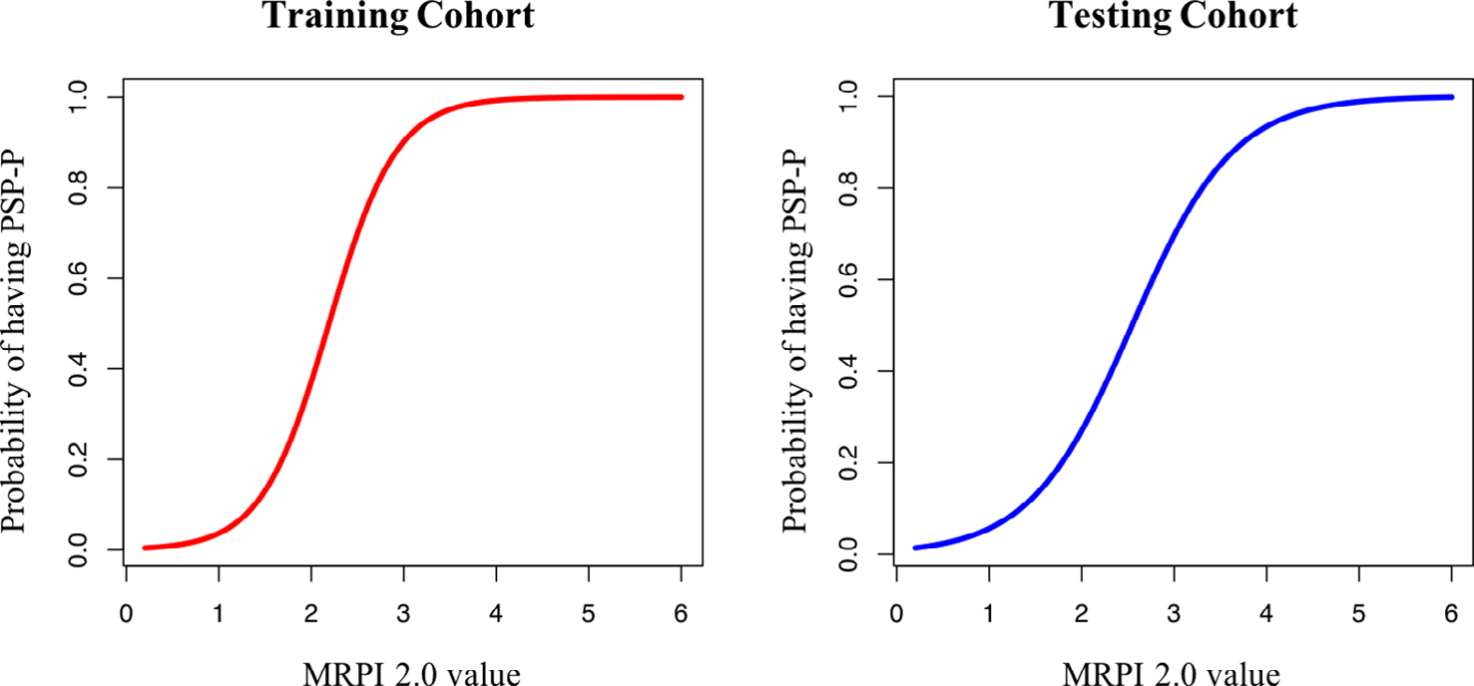
The figure shows the probability of having progressive supranuclear palsy‐parkinsonism (PSP‐P) based on the Magnetic Resonance Parkinsonism Index 2.0 (MRPI 2.0) value in the training (red) and testing (blue) cohorts obtained using logistic regression models. The probability of having PSP‐P increased with higher MRPI 2.0 values in both cohorts. [Color figure can be viewed at wileyonlinelibrary.com]

In both cohorts, automated MRPI 2.0 values were significantly higher in patients with PSP‐P than in patients with PD and control subjects after correcting for age, sex, and MDS‐UPDRS‐III (and scanner field strength in the testing cohort), while no differences were found between patients with PD and control subjects (Table 1). In the PSP‐P group, no significant correlations were found between MRPI 2.0 values and clinical scores (MDS‐UPDRS‐III and H‐Y scores). The automated MRPI 2.0 showed excellent diagnostic performance in distinguishing patients with PSP‐P from patients with PD and control subjects in the training cohort (PSP‐P versus PD: AUC, 0.93; 95% CI, 0.89–0.98; PSP‐P versus controls: AUC, 0.97; 95% CI, 0.93–1.00), and these performances were validated in the international independent testing cohort (PSP‐P versus PD: AUC, 0.92; 95% CI, 0.87–0.96; PSP‐P versus controls: AUC, 0.94; 95% CI, 0.90–0.98) (Fig. 2 and Supporting Information Table S2). The De Long test showed no differences in the diagnostic performance of automated MRPI 2.0 between the training and the testing cohorts (PSP‐P versus PD: D = 0.49, *P* = 0.638; PSP‐P versus controls: D = 0.92, *P* = 0.360), demonstrating the generalizability of our findings (Fig. 2). The accuracy of automated MRPI 2.0 was not affected by the field strength (1.5 or 3 T) of the magnetic resonance (MR) scanner (Supporting Information Table S2). The MRPI 2.0 was also more accurate than MRPI in distinguishing patients with PSP‐P from patients with PD and control subjects, both in the training and the testing cohorts (Supporting Information Fig. S2). Logistic regression analysis showed a strong association between MRPI 2.0 values and PSP‐P diagnosis, suggesting that the higher MRPI 2.0 values, the higher the probability of having PSP‐P (Fig. 3). Moreover, we evaluated the automated MRPI 2.0 in differentiating between patients with PSP‐P and patients with PD in the early stages of the diseases (disease duration up to 3 years). MRPI 2.0 was tested in a subgroup of 37 patients with early PSP‐P (10 from the training and 27 from the testing cohort) and 106 patients with early PD (39 from the training and 67 from the testing cohort) and showed high diagnostic performance (AUC, 0.91; 95% CI, 0.84–0.97) in distinguishing these two early‐stage patient groups (Supporting Information Table S3). MRPI 2.0 outperformed MRPI also when patients with PSP‐P at the early stage of the disease were investigated (Supporting Information Table S3).

To evaluate whether the age difference between PSP‐P and PD might partially contribute to the excellent classification performance of MRPI 2.0, we also performed ROC curve analysis on MRPI 2.0 residuals after correcting for age, showing that the results did not change after age correction. The De Long test showed no differences in the diagnostic performance of automated MRPI 2.0 between ROC curves evaluated on raw MRPI 2.0 values and ROC curves evaluated on residuals (Supporting Information Fig. S3). Notably, male subjects showed higher MRPI 2.0 values than female subjects in each group (*P* < 0.05), and this difference was slightly larger in the PSP‐P group. Thus, the classification accuracy of MRPI 2.0 in distinguishing patients with PSP‐P from patients with PD and control subjects was slightly higher in the male than in the female cohort (Supporting Information Fig. S4). In our study, however, the sex distribution was not statistically different between PSP‐P and PD groups in both cohorts, suggesting that the high classification performance of MRPI 2.0 was due to the disease rather than sex effect.

**FIG 4:**
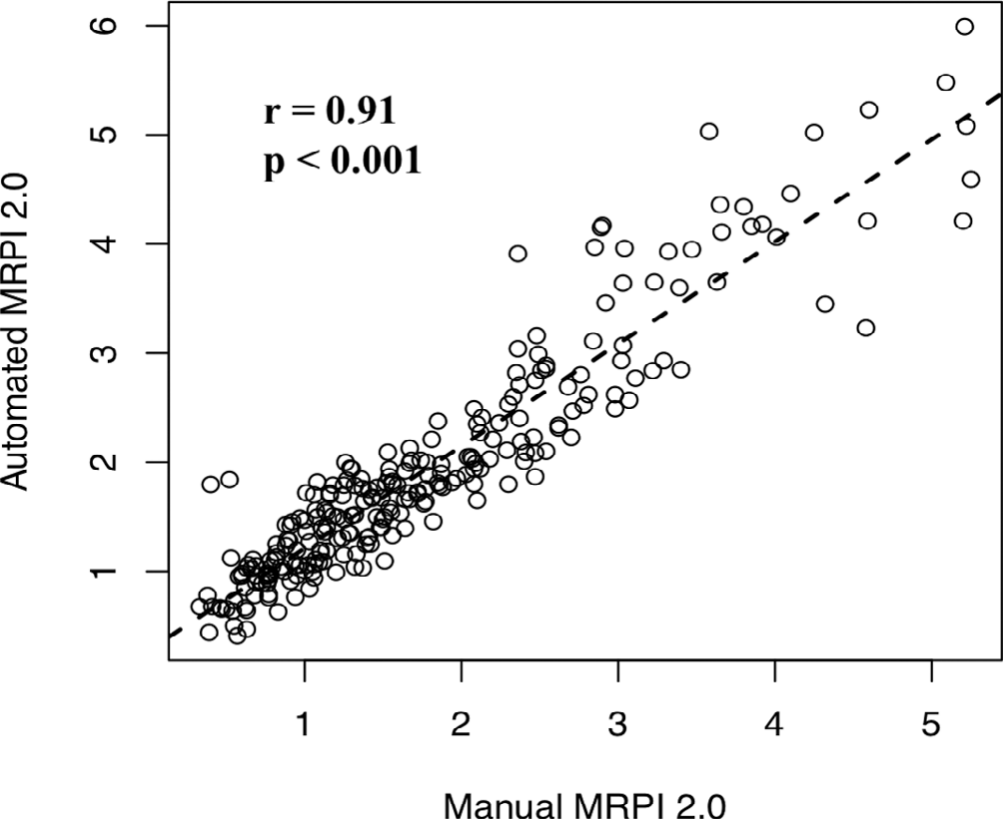
Correlations between automatic and manual Magnetic Resonance Parkinsonism Index 2.0 (MRPI 2.0) values in a subgroup of 250 study participants, including 50 patients with progressive supranuclear palsy‐parkinsonism (PSP‐P), 100 patients with Parkinson's disease (PD), and 100 control subjects.

The automated MRPI 2.0 values showed an excellent correlation with manual measurements (*r* = 0.91; *P* < 0.001) (Fig. 4). Moreover, the automated algorithm showed perfect reproducibility when the whole automated calculation process was repeated twice independently starting from the raw 3D T1‐weighted images (intraclass correlation coefficient = 1).

Finally, we evaluated the diagnostic performance of MRPI 2.0 in an intention‐to‐treat analysis, taking into account the failures of the automated algorithm (Supporting Information Table S4). In brief, we first investigated the classification performance of MRPI 2.0 considering the failures as misclassified patients. As expected, the accuracy was slightly lower (around 84%) in both cohorts (Supporting Information Table S4). Then we investigated the MRPI 2.0 performances when the automated algorithm failed and the MRPI 2.0 values were measured manually. The diagnostic performance of MRPI 2.0 remained very high (AUC ≥ 0.92) in all comparisons when manually measured MRPI 2.0 values were included in the analyses (Supporting Information Table S4).

## Discussion

In this study, we developed an automated algorithm for the MRPI 2.0 calculation and validated its diagnostic performance in differentiating patients with PSP‐P from patients with PD and control subjects in two large independent cohorts from different countries.

In the original description by Steele et al^33^ in 1964, PSP was defined as a progressive disease characterized by vertical gaze and pseudobulbar palsy, nuchal dystonia, and dementia. However, in the last decade, there has been a growing understanding of the PSP clinicopathological spectrum,^2^, ^3^, ^4^, ^5^, ^6^, ^7^, ^8^, ^9^, ^13^, ^34^, ^35^ with many studies reporting the existence of several PSP subtypes other than PSP‐RS. Recently, the MDS has revised the clinical diagnostic criteria for PSP, including international guidelines, to allow a standardized diagnosis of the different PSP variants.^6^

The PSP subtypes other than PSP‐RS, also called PSP variants, represent a considerable percentage of patients with PSP, and several studies reported the PSP‐P as the most frequent one.^2^, ^3^, ^4^, ^5^, ^9^, ^13^ PSP‐P shows a milder severity and slower disease progression than PSP‐RS, associated with a lower degree of brain atrophy detected by MRI and less severe and diffuse tau deposition.^2^, ^3^, ^4^, ^5^, ^10^, ^13^, ^36^, ^37^, ^38^

The correct diagnosis of patients with PSP‐P is challenging also for movement disorder specialists, because these patients often show a clinical phenotype similar to PD, and no validated diagnostic MR imaging biomarkers are currently available to support PSP‐P clinical diagnosis.^2^, ^3^, ^4^, ^5^, ^10^ Indeed, because most diagnostic biomarkers for PSP are based on MRI alterations, which are less marked in PSP variants than in PSP‐RS, powerful biomarkers for PSP‐RS may not show high accuracy for PSP‐P. The MRPI is one of the most widely recognized MRI biomarkers for PSP‐RS.^10^, ^11^, ^12^, ^13^, ^14^, ^15^, ^16^, ^17^, ^18^, ^19^, ^20^, ^21^ However, several studies showed low sensitivity of MRPI in distinguishing PSP‐P from PD, not always meeting the 80% cut point required for an accurate biomarker.^18^, ^22^, ^37^ A recent study in a large international PSP cohort^14^ demonstrated that automated MRPI yielded a very high accuracy in distinguishing patients with PSP‐RS from non‐PSP patients (PD, multiple system atrophy, and control subjects), although it had suboptimal sensitivity in classifying PSP‐P, confirming previous results in this PSP subtype.

To overcome this MRPI limitation in differentiating PSP‐P from PD, because of the less severe atrophy of brainstem structures in PSP‐P than in PSP‐RS, we developed a new version of this biomarker (MRPI 2.0),^22^ which also included in the calculation the 3 V width, a structure that has been widely reported to be enlarged in PSP and spared in PD.^22^, ^23^, ^24^ At the present time, few studies have investigated the diagnostic performance of MRPI 2.0 in distinguishing PSP‐P from PD.^18^, ^22^ In a pilot study from our center,^22^ MRPI 2.0 showed excellent diagnostic accuracy, with high sensitivity and specificity, in distinguishing between these two diseases. Our results, however, were not confirmed in a recent small single‐center study,^18^ which showed high performance of MRPI 2.0 in distinguishing patients with PSP‐P from control subjects but lower accuracy in differentiating patients with PSP‐P from patients with PD, highlighting the need for larger international validation studies. The discrepancy between these studies may be partially related to differences in the manual measurements across centers, which can occur when measuring small brain structures. To standardize MRPI 2.0 measures, in this study we developed an automated algorithm for the MRPI 2.0 calculation and investigated the classification performance of this automated biomarker in two large independent international PSP‐P cohorts. The automated MRPI 2.0 showed an excellent correlation with manual MRPI 2.0 values performed by an expert rater, demonstrating that the new automated algorithm provided reliable measures. In this study, the automated MRPI 2.0 outperformed MRPI and showed excellent diagnostic performance with high sensitivity and specificity (>85%) in differentiating patients with PSP‐P from patients with PD and control subjects in the training cohort (patients with PD: AUC, 0.93; control subjects: AUC: 0.97). Of importance, these findings were validated in the international testing cohort (patients with PD: AUC, 0.92; control subjects: AUC, 0.94), thus suggesting the generalizability of the results.

In addition, this new automated MR biomarker accurately distinguished PSP‐P from PD in a subcohort of patients with short disease duration (up to 3 years from the disease onset), demonstrating its usefulness also in the early stage of the diseases. Our findings in patients with early‐stage PSP are in accordance with previous studies showing that high MRPI 2.0 values predicted the development of PSP clinical features in patients with clinically unclassifiable parkinsonism^39^ or in patients with a clinical diagnosis of PD.^40^ Taken together, these data suggest that MRPI 2.0 is an early diagnostic MR biomarker for PSP, which can also be used to select early‐stage patients for clinical trials with promising disease‐modifying therapies and to predict PSP diagnosis before patients meet clinical criteria. Further longitudinal studies in larger cohorts of early‐stage patients are warranted to confirm these findings.

Overall, this new automated MR algorithm provides reliable and reproducible results significantly improving standardization of the measurements across centers. This finding together with its generalizability makes automated MRPI 2.0 a valid MR biomarker for PSP‐P classification with a positive impact also on research studies and clinical trials involving patients from different geographic regions. The reproducibility guaranteed by automatic measurements also makes this biomarker suitable for longitudinal studies aiming at evaluating disease progression of PSP through repeated measurements over time. A recent study^41^ demonstrated that MRPI 2.0 was able to track disease progression in patients with PSP‐P over 1‐ and 2‐year follow‐up, providing better sample size estimates and effect sizes than clinical scores.

This study has several strengths. First, patients with PSP‐P and PD were from two large and independent cohorts, and the performance of MRPI 2.0 in the training cohort was validated in the international independent cohort, thus ensuring the generalizability of the results. Second, this study involves 99 patients with PSP‐P, representing one of the largest PSP‐P cohorts ever described. Third, we demonstrated that MRPI 2.0 was accurate in distinguishing PSP‐P from PD also in the early stage of the diseases, when the differential diagnosis is much more challenging. Fourth, we developed a reliable fully automated algorithm for MRPI 2.0 calculation to reduce the variability of manual measurements, which need expertise for image reconstruction and slice selection. This software is freely available online (https://mrpi.unicz.it) on registration.

There are some limitations to this study. First, patients with PSP‐P and PD did not undergo a pathological examination; thus, the clinical diagnosis might be in error in a few patients. However, patients with PSP‐P were classified according to international diagnostic criteria for “probable PSP,” which demonstrated high specificity (85.7–91.4%) compared with pathological data.^42^ Future studies confirming the accuracy of MRPI 2.0 in patients with PSP‐P with postmortem examination are warranted. Second, the patients with PSP‐P were older than patients with PD and control subjects; however, we demonstrated that age had no significant effect on MRPI 2.0 diagnostic accuracy. Third, a few patients with PD and control subjects in the testing cohort underwent MRI images with 1.5‐T rather than 3.0‐T scanners. However, the percentage of patients with PD and healthy control subjects correctly classified was similar in the 1.5‐ and 3‐T groups, thus suggesting that a lower MR field strength did not significantly affect the automated MRPI 2.0 calculation.

In conclusion, this study demonstrates that the automated MRPI 2.0 is a powerful, validated, and generalizable MR biomarker for distinguishing PSP‐P from PD in vivo. The automated algorithm for calculating MRPI 2.0 reduces the variability of the measurements across centers, allowing to obtain reliable results worldwide. These findings provide a strong impetus to use automated MRPI 2.0 for supporting clinical diagnosis of PSP‐P, especially in multicentric studies and clinical trials with potential new disease‐modifying therapies.

## Author Roles

1. Research project: A. Conception, B. Organization, C. Execution;

2. Statistical Analysis: A. Design, B. Execution, C. Review and Critique;

3. Manuscript: A. Writing of the first draft, B. Review and Critique;

Andrea Quattrone: 1A, 1B, 1C, 3A, 3B; Maria G. Bianco: 1B, 1C, 2C, 3A; 3B; Angelo Antonini: 1B, 1C, 3B; David E. Vaillancourt: 1B, 1C, 3B; Klaus Seppi: 1B, 1C, 3B; Roberto Ceravolo: 1B, 1C, 3B; Antonio P. Strafella: 1B, 1C, 3B; Gioacchino Tedeschi: 1B, 1C, 3B; Alessandro Tessitore: 1B, 1C, 3B; Roberto Cilia: 1B, 1C, 3B; Maurizio Morelli: 1B, 1C; Salvatore Nigro: 1B, 1C, 3B; Basilio Vescio: 2A, 2B, 2C; Pier Paolo Arcuri: 1B, 1C; Rosa De Micco: 1B, 1C; Mario Cirillo: 1B, 1C; Luca Weis: 1B, 1C; Eleonora Fiorenzato: 1B, 1C; Roberta Biundo: 1B, 1C; Roxana G. Burciu: 1B, 1C; Florian Krismer: 1B, 1C; Nikolaus R. McFarland: 1B, 1C; Christoph Mueller: 1B, 1C; Elke R. Gizewski: 1B, 1C; Mirco Cosottini: 1B, 1C; Eleonora Del Prete: 1B, 1C; Sonia Mazzucchi: 1B, 1C; Aldo Quattrone: 1A, 1B, 3B.

## Full Financial Disclosures for the previous 12 months

A. Antonini has received compensation for consultancy and speaker‐related activities from UCB, Boehringer Ingelheim, Ever Pharma, Jazz Pharmaceuticals, General Electric, Britannia, AbbVie, Kyowa Kirin, Zambon, Bial, Neuroderm, Theravance Biopharma, Roche, and Medscape; he receives research support from Bial, Lundbeck, Roche, Angelini Pharmaceuticals, Horizon 2020 (Grants 825785 and 101016902), Ministry of Education University and Research (MIUR; Grant ARS01_01081), Cariparo Foundation, and Movement Disorder Society for NMS Scale validation. He serves as consultant for Boehringer–Ingelheim for legal cases on pathological gambling.

D.E. Vaillancourt has received grant support from the National Institutes of Health, National Science Foundation, and Tyler's Hope Foundation. He is cofounder and manager of Neuroimaging Solutions, LLC.

K. Seppi reports personal fees from Teva, UCB, Lundbeck, AOP Orphan Pharmaceuticals AG, Roche, Grünenthal, Stada, Licher Pharma, Biogen, and AbbVie; honoraria from the International Parkinson and Movement Disorder Society; and research grants from the FWF Austrian Science Fund, The Michael J. Fox Foundation, and AOP Orphan Pharmaceuticals AG.

R. Ceravolo has received speaking fees from Zambon, UCB Pharma, AbbVie, and Lusofarmaco.

A.P. Strafella is a consultant for Hoffman La Roche and received honoraria from GE Health Care Canada LTD, Hoffman La Roche.

G. Tedeschi has received speaker honoraria from Sanofi Aventis, Merck Serono, Bayer Schering Pharma, Novartis, Biogen‐Dompé AG, Teva, and Lilly; has received funding for travel from Bayer Schering Pharma, Biogen‐Dompé AG, Merck Serono, Novartis, and Sanofi Aventis; and serves as an associate editor of Neurological Sciences.

A. Tessitore has received speaker honoraria from Novartis, Schwarz Pharma/UCB, Lundbeck, AbbVie, and Glaxo.

F. Krismer reports receiving personal fees from Institut de Recherches Internationales Servier, Clarion Healthcare, and the Austrian Society of Neurology and grant support from the MSA Coalition outside of the submitted work.

All other authors report no disclosures.

## Supporting information


**Figure S1** Automated MRPI 2.0 pipeline, from the mid‐sagittal plane identification to automatic segmentation of the third ventricle width and frontal horns width.

**Figure S2** Receiver operating characteristic (ROC) curves for assessing the classification performance of automated MRPI (blue) and MRPI 2.0 (red) in differentiating PSP‐P from PD patients, and PSP‐P from control subjects both in the in the training cohort (A and C) and in the testing cohort (B and D). Abbreviations: MRPI, Magnetic Resonance Parkinsonism Index; PSP‐P, Progressive supranuclear palsy‐parkinsonism; PD, Parkinson's disease; AUC, area under the ROC curve.

**Figure S3** Receiver operating characteristic (ROC) curves for assessing the classification performance of automated MRPI 2.0 in differentiating PSP‐P from PD patients, and PSP‐P from control subjects both in the in the training cohort (A and C) and in the testing cohort (B and D). The red ROC curves were evaluated on raw automated MRPI 2.0 values, while the blue ROC curves were evaluated on residuals after correcting for age. Abbreviations: MRPI 2.0, Magnetic Resonance Parkinsonism Index 2.0; PSP‐P, Progressive supranuclear palsy‐parkinsonism; PD, Parkinson's disease; AUC, area under the ROC curve.

**Figure S4** Receiver operating characteristic (ROC) curves for assessing the classification performance of automated MRPI 2.0 in differentiating PSP‐P from PD patients (A) and control subjects (B) in male (red) and female subjects (blue). De Long test showed no significant differences in the diagnostic performance of automated MRPI 2.0 between the male and female cohorts (PSP‐P vs. PD: D = 1.61, *P* = 0.109; PSP‐P vs. PD: D = 1.36, *P* = 0.175). The male cohort included 66 PSP‐P, 204 PD and 92 controls from both cohorts; the female cohort included 33 PSP‐P, 139 PD and 91 controls from both cohorts. Abbreviations: MRPI 2.0, Magnetic Resonance Parkinsonism Index 2.0; PSP‐P, Progressive supranuclear palsy‐parkinsonism; PD, Parkinson's disease; AUC, area under the ROC curve.

**Table S1** Number of participants for each center.

**Table S2** Diagnostic performance of the Magnetic Resonance Parkinsonism Index 2.0 in differentiating patients with progressive supranuclear palsy‐parkinsonism from those with Parkinson's disease and control subjects, in the training and testing cohorts.

**Table S3** Diagnostic performance of the automated Magnetic Resonance Parkinsonism Index and Magnetic Resonance Parkinsonism Index 2.0 in differentiating between patients with progressive supranuclear palsy‐parkinsonism and those with Parkinson's disease, in the early stage of the diseases.

**Table S4** Diagnostic performance of the Magnetic Resonance Parkinsonism Index 2.0 in differentiating patients with PSP‐P from those with PD and control subjects, also considering the algorithm failures.

**Appendix S1** Supporting Information.

## Data Availability

The data that support the findings of this study are available from the corresponding author upon reasonable request.
