# Supplementary material for: Development and Validation of Automated Magnetic Resonance Parkinsonism Index 2.0 to Distinguish Progressive Supranuclear Palsy‐Parkinsonism From Parkinson's Disease
Source: Mov Disord. 2022 Apr 11;37(6):1272–81. doi: 10.1002/mds.28992 (PMC9321546; doi:10.1002/mds.28992)
Supplement: Supplementary file 1 — Figure S1 Automated MRPI 2.0 pipeline, from the mid‐sagittal plane identification to automatic segmentation of the third ventricle width and frontal horns width. [file MDS-37-1272-s009.pdf]

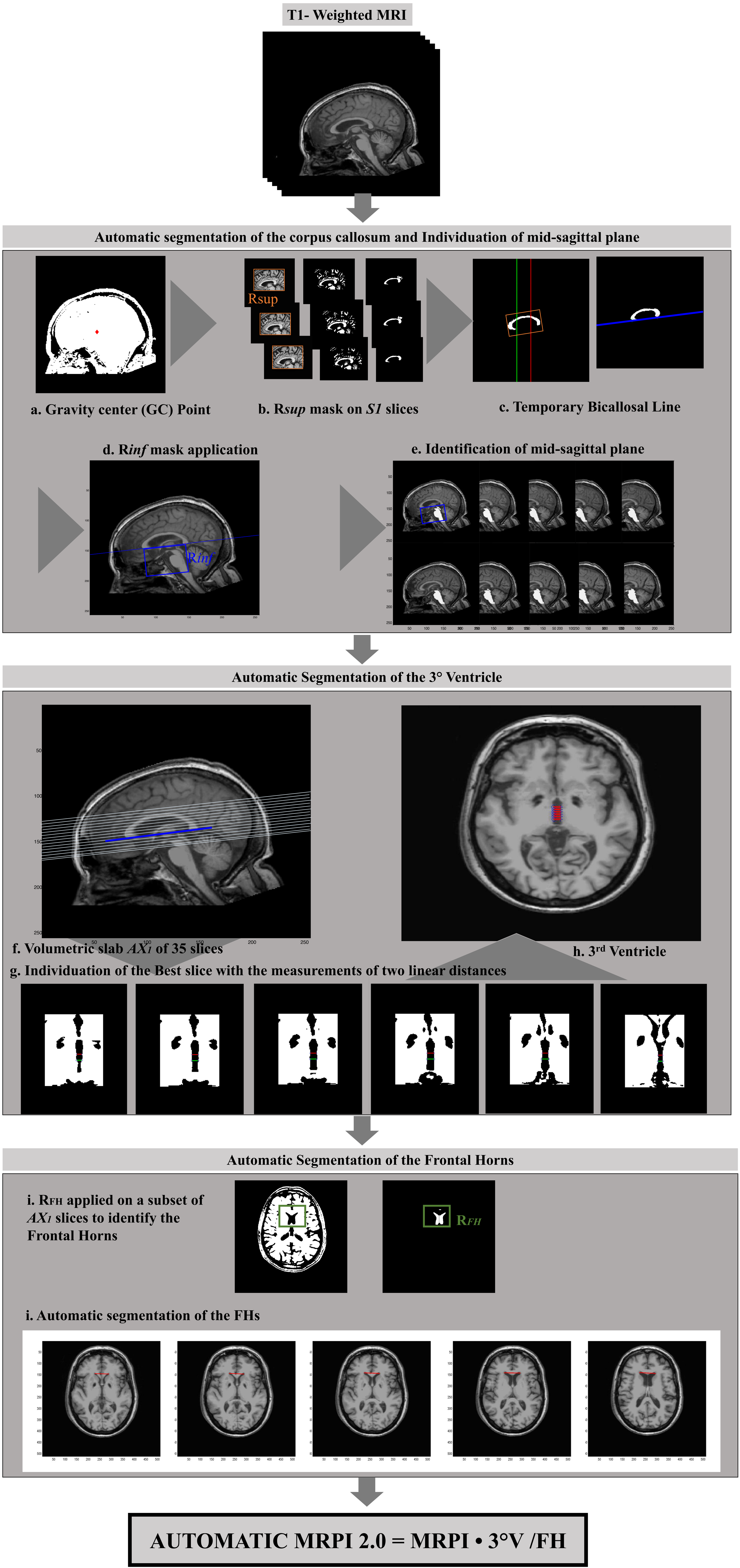
T1- Weighted MRI
Automatic segmentation of the corpus callosum and Individuation of mid-sagittal plane
a. Gravity center (GC) Point
Rsup
b. Rsup mask on S1 slices
c. Temporary Bicallosal Line
d. Rinf mask application
Rinf
e. Identification of mid-sagittal plane
Automatic Segmentation of the 3° Ventricle
f. Volumetric slab AX1 of 35 slices
h. 3rd Ventricle
g. Individuation of the Best slice with the measurements of two linear distances
Automatic Segmentation of the Frontal Horns
i. RFH applied on a subset of AX1 slices to identify the Frontal Horns
RFH
i. Automatic segmentation of the FHs
AUTOMATIC MRPI 2.0 = MRPI • 3°V /FH
