# Supplementary figures and images for: Development and Validation of Automated Magnetic Resonance Parkinsonism Index 2.0 to Distinguish Progressive Supranuclear Palsy‐Parkinsonism From Parkinson's Disease
Source: Mov Disord. 2022 Apr 11;37(6):1272–81. doi: 10.1002/mds.28992 (PMC9321546; doi:10.1002/mds.28992)

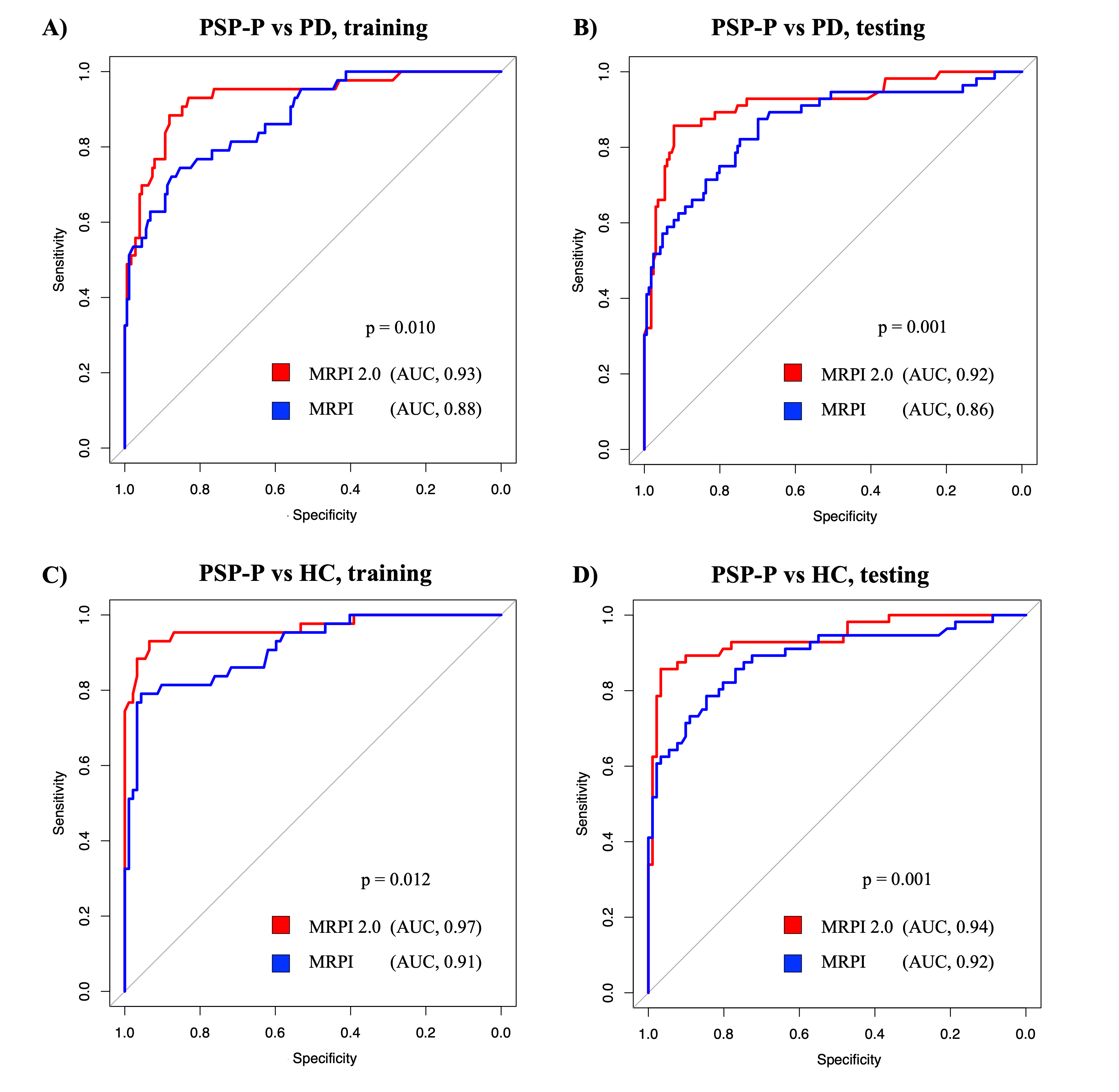

Supplement: Supplementary file 2 — Figure S2 Receiver operating characteristic (ROC) curves for assessing the classification performance of automated MRPI (blue) and MRPI 2.0 (red) in differentiating PSP‐P from PD patients, and PSP‐P from control subjects both in the in the training cohort (A and C) and in the testing cohort (B and D). Abbreviations: MRPI, Magnetic Resonance Parkinsonism Index; PSP‐P, Progressive supranuclear palsy‐parkinsonism; PD, Parkinson's disease; AUC, area under the ROC curve. [file MDS-37-1272-s002.tif]

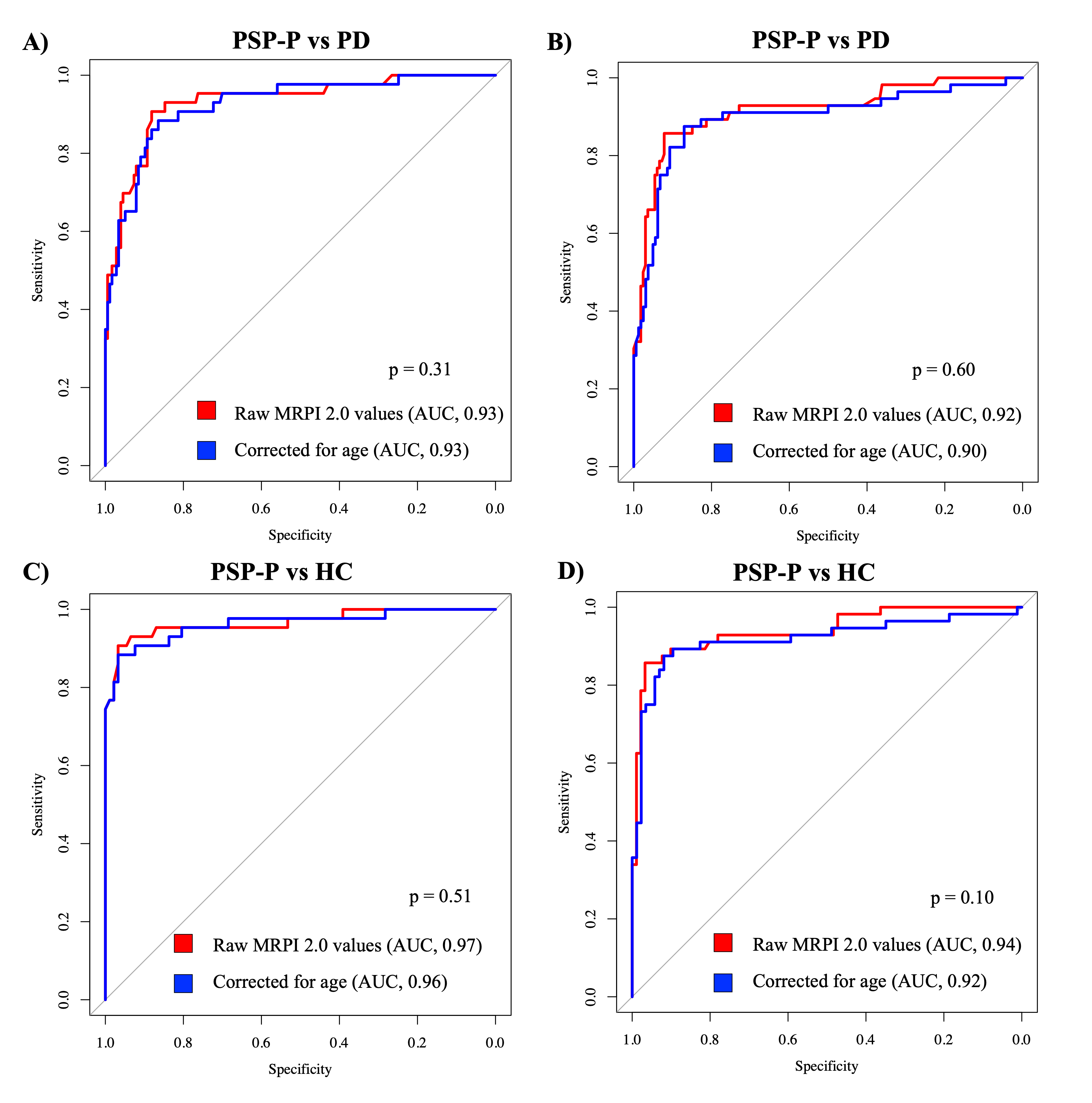

Supplement: Supplementary file 3 — Figure S3 Receiver operating characteristic (ROC) curves for assessing the classification performance of automated MRPI 2.0 in differentiating PSP‐P from PD patients, and PSP‐P from control subjects both in the in the training cohort (A and C) and in the testing cohort (B and D). The red ROC curves were evaluated on raw automated MRPI 2.0 values, while the blue ROC curves were evaluated on residuals after correcting for age. Abbreviations: MRPI 2.0, Magnetic Resonance Parkinsonism Index 2.0; PSP‐P, Progressive supranuclear palsy‐parkinsonism; PD, Parkinson's disease; AUC, area under the ROC curve. [file MDS-37-1272-s007.tif]

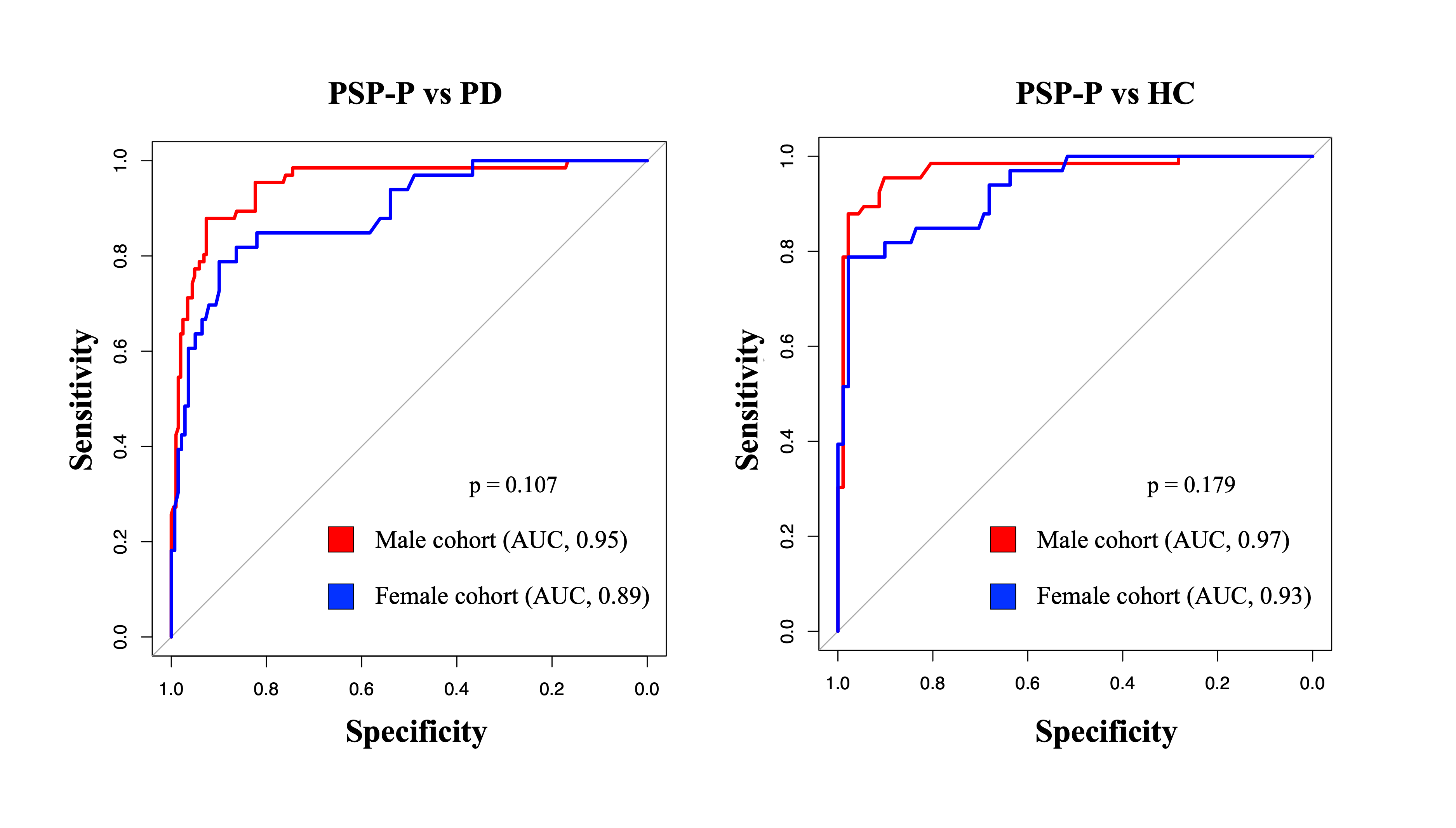

Supplement: Supplementary file 4 — Figure S4 Receiver operating characteristic (ROC) curves for assessing the classification performance of automated MRPI 2.0 in differentiating PSP‐P from PD patients (A) and control subjects (B) in male (red) and female subjects (blue). De Long test showed no significant differences in the diagnostic performance of automated MRPI 2.0 between the male and female cohorts (PSP‐P vs. PD: D = 1.61, P = 0.109; PSP‐P vs. PD: D = 1.36, P = 0.175). The male cohort included 66 PSP‐P, 204 PD and 92 controls from both cohorts; the female cohort included 33 PSP‐P, 139 PD and 91 controls from both cohorts. Abbreviations: MRPI 2.0, Magnetic Resonance Parkinsonism Index 2.0; PSP‐P, Progressive supranuclear palsy‐parkinsonism; PD, Parkinson's disease; AUC, area under the ROC curve. [file MDS-37-1272-s003.tif]
