# Supplementary material for: Development and Validation of Automated Magnetic Resonance Parkinsonism Index 2.0 to Distinguish Progressive Supranuclear Palsy‐Parkinsonism From Parkinson's Disease
Source: Mov Disord. 2022 Apr 11;37(6):1272–81. doi: 10.1002/mds.28992 (PMC9321546; doi:10.1002/mds.28992)
Supplement: Supplementary file 5 — Table S1 Number of participants for each center. [file MDS-37-1272-s001.docx]

**Supplementary Table 1.** Number of participants for each center.

| Center | MR Field strength | PSP-P | PD | HC |
| --- | --- | --- | --- | --- |
| Magna Graecia University^a^ | 3T | 43 (0) | 194 (17) | 109 (17) |
| University of Florida^a^ | 3T | 6 (0) | 43 (0) | 33 (2) |
| Medical University Innsbruck^a^ | 3T | 16 (1) | 39 (1) | 30 (3) |
| University of Toronto^a^ | 3T | - | 5 (0) | - |
| University of Naples^a^ | 3T | 15 (2) | - | - |
| University of Milan^a^ | 3T | 4 (1) | - | - |
| University of Pisa^a^ | 1.5T and 3T | 10 (2) | 37 (1) | 5 (0) |
| University of Padua^a^ | 1.5T and 3T | 11 (1) | 47 (2) | 29 (1) |

Abbreviations: PSP-P=progressive supranuclear palsy-parkinsonism; PD = Parkinson's disease; HC = Healthy Controls; T = Tesla. ^a^Data are presented as number of participants (number of failures)
