# Supplementary material for: Development and Validation of Automated Magnetic Resonance Parkinsonism Index 2.0 to Distinguish Progressive Supranuclear Palsy‐Parkinsonism From Parkinson's Disease
Source: Mov Disord. 2022 Apr 11;37(6):1272–81. doi: 10.1002/mds.28992 (PMC9321546; doi:10.1002/mds.28992)
Supplement: Supplementary file 6 — Table S2 Diagnostic performance of the Magnetic Resonance Parkinsonism Index 2.0 in differentiating patients with progressive supranuclear palsy‐parkinsonism from those with Parkinson's disease and control subjects, in the training and testing cohorts. [file MDS-37-1272-s008.docx]

**Supplementary Table 2.** Diagnostic performance of the Magnetic Resonance Parkinsonism Index 2.0 in differentiating patients with progressive supranuclear palsy-parkinsonism from those with Parkinson’s disease and control subjects, in the training and testing cohorts.

|  | **MRPI 2.0** | |
| --- | --- | --- |
| **Cut-off and statistical values** | **Training cohort** | **Testing cohort** |
| ***PSP-P patients vs PD patients*** |  |  |
| Cutoff value | ≥ 2.23 (2.08-2.35) | ≥ 2.70 (2.42-2.95) |
| Sensitivity (%) | 93.0 (83.7-100) | 85.7 (76.8-94.6) |
| Specificity (%) | 87.6(80.8-92.3) | 92.2 (86.1-96.4) |
| Accuracy (%) | 88.6 (83.2-93.2) | 90.5 (86.0-94.1) |
| AUC (%) | 0.93 (0.89-0.98) | 0.92 (0.87-0.96) |
| ***PSP-P patients vs control subjects*** |  |  |
| Cutoff value | ≥ 2.21 (1.76-2.28) | ≥ 2.74 (2.30-2.89) |
| Sensitivity (%) | 93.0 (83.7-100) | 87.5 (76.8-94.6) |
| Specificity (%) | 95.6 (89.1-100) | 96.7 (90.1-100) |
| Accuracy (%) | 94.8 (90.4-98.5) | 92.5 (87.8-96.6) |
| AUC (%) | 0.97 (0.93-1.00) | 0.94 (0.90-0.98) |

Abbreviations: PSP-P = Progressive supranuclear palsy-parkinsonism; PD = Parkinson’s disease; MRPI 2.0 = Magnetic Resonance Parkinsonism Index 2.0; AUC = area under the curve. PSP-P was considered a positive finding; PD and controls were considered negative findings. The testing cohort included 43 participants (26 PD patients and 17 control subjects) who underwent a 1.5T MR examination, and 39 of these 43 subjects were correctly classified using MRPI 2.0, demonstrating that a lower MR field strength does not affect the automated MRPI 2.0 accuracy.
