# Supplementary material for: Development and Validation of Automated Magnetic Resonance Parkinsonism Index 2.0 to Distinguish Progressive Supranuclear Palsy‐Parkinsonism From Parkinson's Disease
Source: Mov Disord. 2022 Apr 11;37(6):1272–81. doi: 10.1002/mds.28992 (PMC9321546; doi:10.1002/mds.28992)
Supplement: Supplementary file 7 — Table S3 Diagnostic performance of the automated Magnetic Resonance Parkinsonism Index and Magnetic Resonance Parkinsonism Index 2.0 in differentiating between patients with progressive supranuclear palsy‐parkinsonism and those with Parkinson's disease, in the early stage of the diseases. [file MDS-37-1272-s005.docx]

**Supplementary Table 3.** Diagnostic performance of the automated Magnetic Resonance Parkinsonism Index and Magnetic Resonance Parkinsonism Index 2.0 in differentiating between patients with progressive supranuclear palsy-parkinsonism and those with Parkinson’s disease, in the early stage of the diseases.

| **Cut-off and statistical values** | **MRPI** | **MRPI 2.0** |
| --- | --- | --- |
| ***Early PSP-P patients vs early PD patients*** |  |  |
| Cutoff value | ≥ 12.50 (9.89-14.46) | ≥ 2.85 (1.76-3.32) |
| Sensitivity (%) | 78.4 (59.5-100) | 81.1 (64.9-97.3) |
| Specificity (%) | 84.0 (55.7-97.2) | 94.3 (72.6-100) |
| Accuracy (%) | 82.5 (66.4-89.5) | 90.2 (77.6-95.1) |
| AUC | 0.87 (0.80-0.94) | 0.91 (0.84-0.97) |

Abbreviations: PSP-P = Progressive supranuclear palsy-parkinsonism; PD = Parkinson’s disease; MRPI = Magnetic Resonance Parkinsonism Index; AUC = area under the curve. All PSP-P and PD patients had disease duration within three years from the disease onset. PSP-P was considered to be a positive finding; PD was considered to be a negative finding. The early stage cohort included 37 early PSP-P patients (10 from the training and 27 from the testing cohort) and 106 early PD patients (39 from the training and 67 from the testing cohort).
