# Supplementary material for: Development and Validation of Automated Magnetic Resonance Parkinsonism Index 2.0 to Distinguish Progressive Supranuclear Palsy‐Parkinsonism From Parkinson's Disease
Source: Mov Disord. 2022 Apr 11;37(6):1272–81. doi: 10.1002/mds.28992 (PMC9321546; doi:10.1002/mds.28992)
Supplement: Supplementary file 8 — Table S4 Diagnostic performance of the Magnetic Resonance Parkinsonism Index 2.0 in differentiating patients with PSP‐P from those with PD and control subjects, also considering the algorithm failures. [file MDS-37-1272-s006.docx]

**Supplementary Table 4.** Diagnostic performance of the Magnetic Resonance Parkinsonism Index 2.0 in differentiating patients with PSP-P from those with PD and control subjects, also considering the algorithm failures.

|  | **MRPI 2.0** | |
| --- | --- | --- |
| **Cut-off and statistical values** | **Training cohort** | **Testing cohort** |
| ***Algorithm failures were considered as misclassificated cases*** |  |  |
| ***PSP-P patients vs PD patients*** |  |  |
| Cutoff value | ≥ 2.23 | ≥ 2.70 |
| Sensitivity (%) | 90.7 | 75.8 |
| Specificity (%) | 80.4 | 90.0 |
| Accuracy (%) | 82.3 | 86.3 |
|  |  |  |
| ***PSP-P patients vs control subjects*** |  |  |
| Cutoff value | ≥ 2.21 | ≥ 2.74 |
| Sensitivity (%) | 90.7 | 75.8 |
| Specificity (%) | 81.7 | 89.7 |
| Accuracy (%) | 84.2 | 84.3 |
| ***In algorithm failures, the MRPI 2.0 were measured manually*** |  |  |
| ***PSP-P patients vs PD patients*** |  |  |
| Cutoff value | ≥ 2.23 (2.08-2.35) | ≥ 2.70 (2.64-2.91) |
| Sensitivity (%) | 93.0 (83.7-100) | 86.0 (75.4-94.7) |
| Specificity (%) | 87.6 (81.4-93.3) | 92.3 (87.6-96.5) |
| Accuracy (%) | 88.6 (83.5-92.8) | 90.7 (86.3-94.3) |
| AUC (%) | 0.93 (0.89-0.98) | 0.92 (0.87-0.97) |
|  |  |  |
| ***PSP-P patients vs control subjects*** |  |  |
| Cutoff value | ≥ 2.21 (1.77-2.35) | ≥ 2.74 (2.30-2.89) |
| Sensitivity (%) | 93.0 (83.7-100) | 87.7 (78.9-96.5) |
| Specificity (%) | 96.3 (89.9-99.1) | 96.9 (89.7-100) |
| Accuracy (%) | 94.7 (90.1-98.0) | 92.9 (89.0-96.7) |
| AUC (%) | 0.97 (0.93-1.00) | 0.94 (0.90-0.98) |

Abbreviations: PSP-P = Progressive supranuclear palsy-parkinsonism; PD = Parkinson’s disease; MRPI 2.0 = Magnetic Resonance Parkinsonism Index 2.0; AUC = area under the curve. PSP-P was considered a positive finding; PD and controls were considered negative findings. The training cohort included 43 PSP-P, 194 PD and 109 controls from our centre; the independent testing cohort included 62 PSP-P, 171 PD and 97 controls from an international research group.
