# Supplementary material for: Development and Validation of Automated Magnetic Resonance Parkinsonism Index 2.0 to Distinguish Progressive Supranuclear Palsy‐Parkinsonism From Parkinson's Disease
Source: Mov Disord. 2022 Apr 11;37(6):1272–81. doi: 10.1002/mds.28992 (PMC9321546; doi:10.1002/mds.28992)
Supplement: Supplementary file 9 — Appendix S1 Supporting Information. [file MDS-37-1272-s004.docx]

**Supplementary Materials**

*Pipeline for the automated MRPI 2.0 calculation*

The T1-weighted brain 3T MR images were acquired via a web-based framework as previously described.^1^ The MR images were rigidly registered to the Montreal Neurological Institute space (MNI) using a 6-parameter affine registration with FSL software (FMRIB Software Library). Intensity normalization of the volumes was performed to corrects drop out in the images with FreeSurfer software package.^1,2^ The previously described toolbox for automated MRPI calculation,^3^ followed by the new algorithm for MRPI 2.0 calculation, was applied to anonymized DICOM files (Supplementary Figure 1). The proposed segmentation framework consisted of a combined deterministic approach based on anatomical landmarks and thresholding-based methods.^4^ In detail, the algorithm for the automated MRPI 2.0 calculation consisted of the following three steps:

1. *Automated segmentation of the corpus callosum and individuation of mid-sagittal plane adopting a modified version of Nigro’s algorithm.^3^*

The middle slice of T1-weighted images was binarized using Otsu's thresholding method.^5^ This thresholding technique separated into two classes (foreground and background) each pixel according to image histogram. The method binarized the image objects minimizing the variance on each of the classes (global threshold *t1+0.30*). The flood fill algorithm was used to exact features from a binary slice labelling all the 8-connected pixels. The centroid of the binarized brain was returned as gravity center (GC) point, as shown in Figure S1a.

As proposed by Nigro et al,^3^ a subgroup of 20 slices (*S1*) centered on the middle slice of overall volume, was chosen to segment the corpus collosum (CC). A region of interest (R-superior, R*sup,* Figure S1b) was defined on the sagittal plane from the GC, considering the inclination of the head. The modification in the previously described algorithm^3^ consisted of using a rectangular mask adaptable to the orientation of the head, to include the CC in the region of interest. Subsequently, the image was enhanced in the contrast by using the contrast-limited adaptive histogram equalization (CLAHE), with a 0.02 contrast enhancement limit of 64 tiles,^6^ binarized (Otsu’s method) and image denoised, excluding pixel out of the region R*sup* and including those with the biggest connected components (8-connected components in each binary slice with flood fill algorithm). The slice with the smallest area of the corpus callosum was considered in order to define a temporary sub-callosal line. The CC was divided into four regions taking in account its bounding box and the GC coordinates. The algorithm identified the Genu/Rostrum and Splenium as anatomical landmarks to draw a temporary subcallosal line (Figure S1c). This reference was used to realize an adaptive mask R-inferior (R*inf,* Figure S1d) to identify the upper part of the brainstem. The mid-sagittal slice was identified as the slice with a minimal area in upper part of the brainstem and the maximal expansion of the Sylvius aqueduct, as described by Nigro et al.^3^ Once identified the mid-sagittal slice using anatomical markers as reference, the sub-callosal line was traced again using the procedure described above (Figure S1e).

2) *Segmentation of the 3^rd^ ventricle and Frontal Horns of the lateral ventricles*.

By using T1-weighted images, a reformatted volumetric slab of 35 axial slices (*AX_1_*) parallel to the subcallosal line was generated to expose the 3^rd^ ventricle (3V) and the frontal horns (FH) of the lateral ventricles (Figure S1f). In detail, the binarization (threshold level= t1 + 0.10 where t1 is the global threshold calculated with Otsu’s method) and enhancement of contrast (CLAHE function) were applied on each slice of *AX1* slab. At this point, in each axial slice showing the 3V, the algorithm performed two automated linear measurements between its lateral borders and identified the slice with the largest 3V width (Figure S1g). Subsequently, in this selected slice, the 3V width was calculated as the mean of six automated linear measures of the distance between its lateral borders, and this value was used for MRPI 2.0 calculation (Figure S1h).

As previously described,^7^ the frontal horns were evaluated on axial slices at the level of their maximal dilation. A mask (Region Frontal Horns R*fh)* was placed on the frontal lobe in a subset of 15 slices of the volumetric *AX_1_* slab. Otsu’s method for binarization (*t1*+0.10) and contrast enhancement (16 tiles) were used as pre-processing step, and the left-to-right distance between the lateral borders of the frontal horns was measured in each slice. The maximum value was used for the MRPI 2.0 calculation (Figure S1i).

*3) Automated MRPI 2.0 calculation*

The Automated MRPI 2.0 value was obtained multiplying the MRPI value (calculated using the method previously described)^3^ by the value of the automated 3V width / FH width ratio, as previously described.^7^

**Supplementary References**

1. Nigro S, Antonini A, Vaillancourt DE, et al. Automated MRI Classification in Progressive Supranuclear Palsy: A Large International Cohort Study. Mov Disord. 2020; 35(6):976-983. doi: 10.1002/mds.28007.
2. Dale AM, Fischl B, Sereno MI. Cortical surface-based analysis. I. Segmentation and surface reconstruction. Neuroimage. 1999; 9(2):179-94. doi: 10.1006/nimg.1998.0395.
3. Nigro S, Arabia G, Antonini A, et al. Magnetic Resonance Parkinsonism Index: diagnostic accuracy of a fully automated algorithm in comparison with the manual measurement in a large Italian multicentre study in patients with progressive supranuclear palsy. Eur Radiol. 2017; 27(6):2665-2675. doi: 10.1007/s00330-016-4622-x.
4. Nigro S, Cerasa A, Zito G, et al. Fully automated segmentation of the pons and midbrain using human T1 MR brain images. PLoS One. 2014; 9(1):e85618. doi: 10.1371/journal.pone.0085618.
5. N. Otsu, "A Threshold Selection Method from Gray-Level Histograms," in IEEE Transactions on Systems, Man, and Cybernetics. vol. 9, no. 1, 62-66, Jan. 1979, doi: 10.1109/TSMC.1979.4310076.
6. Zuiderveld K. Contrast Limited Adaptive Histograph Equalization. Graphic Gems IV. 1994: 474–485. doi: 10.1016/b978-0-12-336156-1.50061-6
7. Quattrone A, Morelli M, Nigro S, et al. A new MR imaging index for differentiation of progressive supranuclear palsy-parkinsonism from Parkinson's disease. Parkinsonism Relat Disord. 2018; 54: 3-8. doi: 10.1016/j.parkreldis.2018.07.016.
